# Intratumoral microbiota remodeling of the tumor microenvironment impact solid tumor immunotherapy

**DOI:** 10.1038/s41419-025-08211-w

**Published:** 2025-12-01

**Authors:** Jiawen Jia, Wenwen Gao, Cong Wang, Ling Liu, Yiqian Li, Zhenqiang Sun, Cuiping Yang, Yongbin Chen, Haibin Yu, Yan Cheng

**Affiliations:** 1 Department of Gynecological Oncology Radiotherapy, The First Affiliated Hospital of Zhengzhou University, Zhengzhou City, Henan Province China; 2 Department of Colorectal Surgery, The First Affiliated Hospital of Zhengzhou University, Zhengzhou City, Henan Province China; 3 The International Peace Maternity and Child Health Hospital, School of Medicine, Shanghai Jiao Tong University, Shanghai City, China; 4 Shanghai Key Laboratory of Embryo Original Diseases, Shanghai City, China; 5 The First Affiliated Hospital of Zhengzhou University, Zhengzhou City, Henan Province China; 6 Key Laboratory of Animal Models and Human Disease Mechanisms of Chinese Academy of Sciences & Yunnan Province, Kunming Institute of Zoology, Kunming City, Yunnan Province China; 7 Department of Intervention, The Second Affiliated Hospital of Zhengzhou University, Zhengzhou City, Henan Province China

**Keywords:** Immunization, Cancer immunotherapy

## Abstract

Tumor immunotherapy has significantly advanced in recent years. However, few patients with solid tumors respond to immunotherapy. The tumor microenvironment (TME) is a key factor in immunotherapy efficacy, and the intratumoral microbiota plays a significant role in remodeling the TME. Recent multiomic analyses revealed bacterial signatures in up to 76% of pancreatic ductal adenocarcinoma (PDAC) cases, and specific microbial consortia were linked to therapeutic resistance. Microbiota-targeted therapies, such as engineered bacterial strains, increase tumor clearance rates by approximately 30% in preclinical models. However, the complexity of microbial mechanisms and heterogeneity among individuals limit the clinical translation of intratumoral microbiota-based therapies. In this review, we provide an overview of intratumoral microorganisms, explore their influence on immune cells and signaling pathways in the TME, and discuss their potential value for improving the response of solid tumors to immunotherapy. Specifically, we examine the unique characteristics of intratumoral microbes in different types of solid tumors, emphasizing how tumor microsatellite status plays a key role in determining the effects of intratumoral microbes on the response of solid tumors to immunotherapy. Additionally, we discuss the clinical application of the intratumoral microbiota as a potential method for improving treatment efficacy and prognosis prediction in patients receiving immunotherapy for solid tumors.

## Facts

- Immunotherapy represents a significant advancement in cancer treatment. However, immunotherapy efficacy in solid tumors is often constrained by complex immune interactions in the tumor microenvironment.
- The biological significance of the intratumoral microbiota has been extensively studied and has profound implications for cancer diagnosis and treatment.
- The intratumoral microbiota can be exploited as a therapeutic target, biomarker, and platform for drug delivery, and specific bacteria may be used as biological agents.
- Although intratumoral microbiota-based strategies have improved the efficacy of anticancer immunotherapies for solid tumors, numerous challenges remain regarding their clinical translation.

## Open questions

- How does the intratumoral microbiota modulate the tumor microenvironment (TME) to affect immunotherapy efficacy in solid tumors?
- How does the intratumoral microbiota in different solid tumor types affect the efficacy of cancer immunotherapy?
- What are clinical applications for the intratumoral microbiota in immunotherapy for solid tumors?
- How can the challenges in the clinical translation of the intratumoral microbiota in solid tumor immunotherapy be overcome?

## Introduction

Immunotherapy is beneficial for treating solid tumors [1], but its efficacy remains unsatisfactory. Hence, researchers are exploring ways to increase the sensitivity of solid tumors to immunotherapy. One area of research is the intratumoral microbiota [2], which comprises all microorganisms in the tumor microenvironment (TME), including bacteria, archaea, fungi, viruses, and other microorganisms that contribute to microenvironmental remodeling [3]. The microbiota profile is highly specific for each tumor type. These microbial communities can enhance antitumor immunity and immunotherapy outcomes by activating the STING pathway, T cells, and NK cells, as well as by generating Tertiary lymphoid structures (TLSs) and presenting antigens. Alternatively, the microbiota can suppress the antitumor immune response by increasing ROS levels, fostering an anti-inflammatory environment, deactivating T cells, and promoting immunosuppression [4, 5].

However, many critical questions, such as how spatial heterogeneity affects immunomodulatory activities and how the microbiota controls immune checkpoint inhibitor (ICI) responsiveness, remain unsolved [2, 6, 7]. The link between microbiome alterations during immunotherapy and TME remodeling or resistance is also being investigated. Furthermore, targeting the intratumoral microbiome while preserving the commensal gut microbiota remains a challenge [6, 8]. Addressing these challenges may clarify the role of the intratumoral microbiota in the response to cancer immunotherapy and facilitate the development of more effective treatment strategies. Therefore, investigating the mechanisms by which the intratumoral microbiota regulates the TME, such as by promoting the recruitment and activation of immune cells and related stromal components, is critical to improve the treatment response and prognosis of patients with solid tumors.

This review explores the composition of intratumoral microbes, their roles in modulating the TME of solid tumors, and the probable mechanisms and routes through which they affect immunotherapy efficacy. Furthermore, we outline the current limits of the intratumoral microbiota for application in immunotherapy for solid tumors and highlight future possibilities.

## Heterogeneity and classification of the intratumoral microbiota

**Fig. 1:**
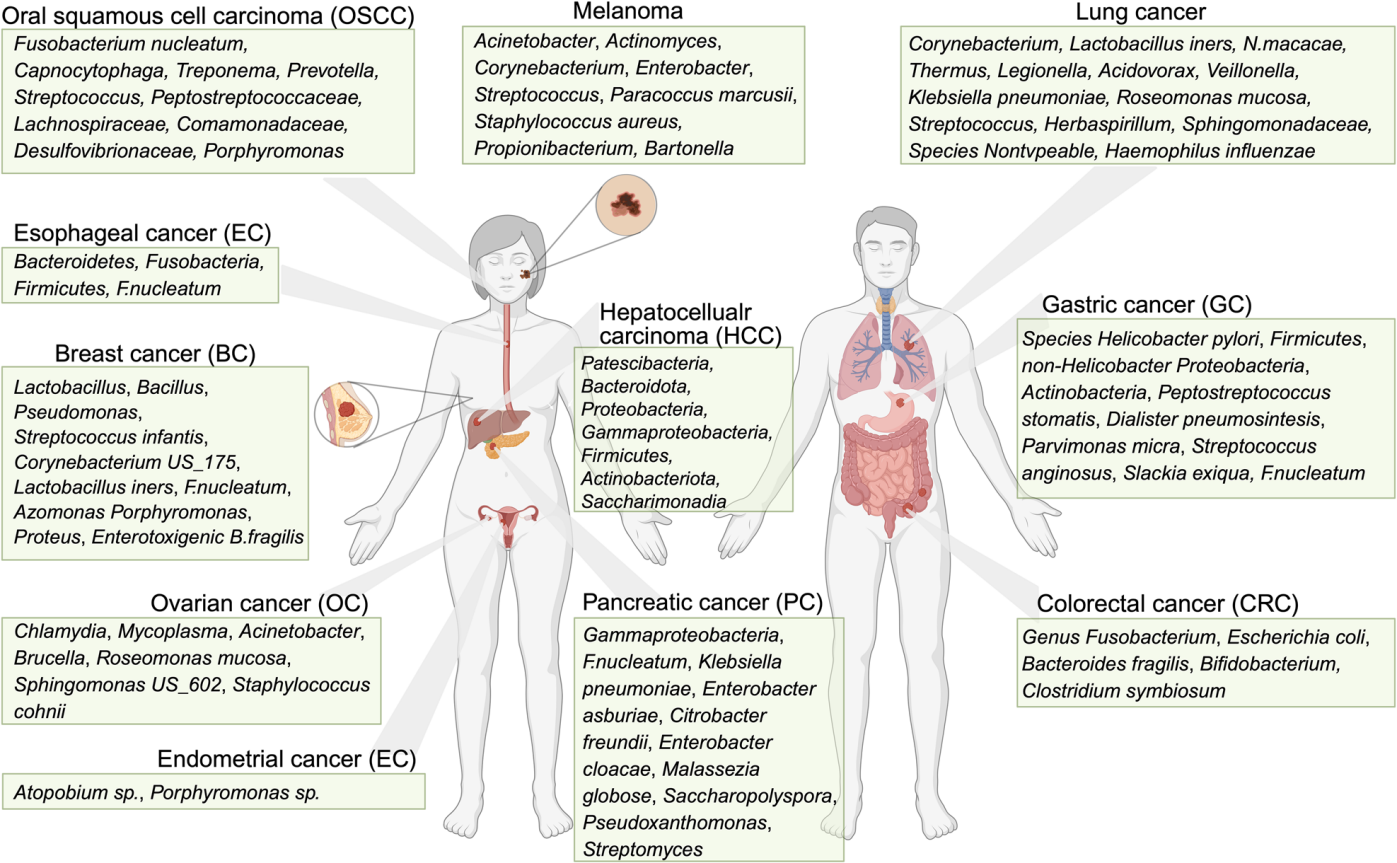
Tumor-type specific intratumoral microbiota associations across human organs. The intratumoral microbiota demonstrates distinct compositional profiles across organ-specific tumor types. For example, tumors of the oral cavity and gastrointestinal tract, such as oral squamous cell carcinoma (OSCC), esophageal cancer, gastric cancer (GC), colorectal cancer (CRC) and pancreatic cancer (PC), are enriched with bacteria commonly found in the digestive tract, including *Fusobacterium nucleatum* (*F. nucleatum*). GC tissues are strongly associated with *Helicobacter pylori* enrichment. Other cancer forms, such as melanoma, lung cancer, hepatocellular carcinoma (HCC), ovarian cancer (OC), and breast cancer (BC), also have distinct microbial communities that reflect the anatomical and microenvironmental circumstances. Created with BioRender.com.

The intratumoral microbiota, an important component of the TME, shows tumor type-specific heterogeneity. Analysis of nearly 1500 clinical samples revealed diverse microbial compositions across various malignancies, including lung, ovarian, pancreatic, breast, melanoma, and brain tumors [9]. The components, enrichment levels and functions of the intratumoral microbiota in specific cancer types are shown in Tables 1, 2 and Fig. 1. The features of the microbiota vary among patients grouped by tumor subtype, stage, and survival outcome, thus highlighting their diagnostic and prognostic value [9]. Intracellular bacteria account for most of the tumor microbiota. For example, *Thermus* and *Legionella* bacteria are abundant in advanced and metastatic tumors, respectively. The tumor microbiomes of long-term cancer survivors are more diverse than those of short-term cancer survivors and are enriched in specific survival-related bacteria [10]. Notably, the proportion of bacterial DNA-positive tumors varies from 14.3% among melanomas to more than 60% among pancreatic, breast, and bone cancers [9].

**Table 1:** Characterization and function of the intratumoral microbiota in gastrointestinal cancers.

Tumor type	Changes	Intratumoural microbiota	Function
Gastrointestinal cancers			
Esophageal cancer	Increase	*Bacteroidetes, Fusobacteria, Firmicutes* [145]	
		*F. nucleatum* [146]	Poor prognosis
	Decrease	*Proteobacteria*	
GC	Increase	*Species Helicobacter pylori* [72, 147]	Activates CEACAM1 (immune cell)
			Induces PD-L1 expression
		*Firmicutes*, *non-Helicobacter Proteobacteria*, *Actinobacteria* [145]	
		*Peptostreptococcus stomatis*, *Dialister pneumosintesis Parvimonas* *micra*, *Streptococcus anginosus*, *Slackia exigua* [148],	Related to tumor pregression
	Decrease	*Helicobacter pylori* [145]	
HCC	Increase	*Patescibacteria, Bacteroidota, Proteobacteria* [149]	
		*Gammaproteobacteria, Firmicutes, Actinobacteriota*, *Saccharimonadia* [149]	Related to tumor progression or prognosis
PC	Increase	*Gammaproteobacteria* [150]	Influence anti-tumor immunity and chemotherapy
		*F. nucleatum*, *Klebsiella pneumoniae*, *Enterobacter asburiae*, *Citrobacter freundii*,	
		*Enterobacter cloacae* [9],	
		*Malassezia globose* [151],	Activates complement pathway/ induces tumorigenesis
		*Saccharopolyspora, Pseudoxanthomonas, Streptomyces* [10]	Associated with increased long- term survival
CRC	Increase	*Genus Fusobacterium* [64, 152, 153]	Promotes M2 macrophage differentiation
		*Clostridium symbiosum* [9]	
		*Escherichia coli, Bacteroides fragilis* [142]	Protective against metastases (inhibits GVB)
		*Bifidobacterium* [94]	Activates anti-tumor immunity (STING signaling)
	Heterogeneous	*Bacteroides, Parvimonas, Prevotella* [154, 155]	

*GC* gastric cancer, *HCC* hepatocellular carcinoma, *PC* pancreatic cancer, *CRC* colorectal cancer, *CEACAM1* carcinoembryonic antigen-related cell adhesion molecule 1, *PD-L1* programmed cell death ligand 1, *GVB* gut vascular barrier, *STING* Stimulator of Interferon Genes.

**Table 2:** Characterization and function of the intratumoral microbiota in non-gastrointestinal cancers.

Tumor type	Changes	Intratumoural microbiota	Function
Head and Neck Cancers			
HNSCC	Increase	*Parvimonas* [156]	Related to the tumor progression
	Decrease	*Actinomyces* [156]	
OSCC	Increase	*Capnocytophaga* [157],	Impairs anti-tumor T cell responses (memory T cells)
		*Fusobacterium nucleatum* [158]	
		*Treponema* [157], *Prevotella, Peptostreptococcaceae*[157], *Leptotrichia, Oscillospira, Pseudomonas*, *Lachnospiraceae, Comamonadaceae, Desulfovibrionaceae* [157]	Impairs anti-tumor T cell responses (immunosuppressive IL-10/GATA3^+^ T cells)
		*Porphyromonas* [159]	Related to tumor progression or prognosis
Thoracic Tumors			
Lung cancer	Increase	*Corynebacterium, Neisseria macacae, Klebsiella pneumoniae*, *Lactobacillus iners, Roseomonas mucosa* [9]	
		*Thermus, Legionella* [160]	
		*Acidovorax* [161],	Associated with high TP53 mutation
		*Veillonella, Streptococcus* [162, 163]	Activates pro-oncogenic pathways (ERK, PI3K)
		*Herbaspirillum, Sphingomonadaceae* [44]	Activates γδ T cells
		*Species Nontypeable Haemophilus influenzae* [164]	Recruits neutrophils / IL-17C release
	Decrease	*Aggregatibacter, Lactobacillus* [44]	
BC	Increase	*Bacillus, Pseudomonas* [165]	
		*Streptococcus infantis, Corynebacterium US_175, Lactobacillus iners* [166, 167]	
		*F. nucleatum* [98]	Increases myeloid cell infiltration (an Immunosuppressive TME)
		*Azomonas Porphyromonas, Proteus* [166]	
		*Enterotoxigenic B. fragilis* [168]	Promotes tumorigenesis (Notch1/β-catenin activation via BFT)
	Decrease	*Propionibacterium Staphylococcus* [166]	Protective role suggested (Microbial)
Gynecologic Oncology			
OC	Increase	*Chlamydia, Mycoplasma, Acinetobacter, Brucella* [169]	Contributes to direct/indirect tumorigenesis
		*Roseomonas mucosa, Sphingomonas US_602, Staphylococcus cohnii* [9, 169]	
EC	Increase	*Atopobium sp. Porphyromonas sp*. [170]	
Skin cancers			
melanoma	Increase	*Acinetobacter, Actinomyces, Corynebacterium, Enterobacter*, *Streptococcus* [102]	
		*Paracoccus marcusii, Staphylococcus aureus* [171]	
		*Propionibacterium, Bartonella* [9]	
NMSC	Increase	*Staphylococcus aureus* [171]	Significant in SCC / associated with carcinogenesis
	Decrease	*Malassezia* [171]	
		*Staphylococcus epidermidis* [172]	Anti-tumor via release of 6-HAP

*HNSCC* head and neck squamous cell carcinoma, *OSCC* oral squamous cell carcinoma, *BC* breast cancer, *OC* ovarian cancer, *EC* endometrial cancer, *NMSC* non-melanoma skin cancers, *TILs* tumor infiltrating lymphocytes, *TME* the tumor microenvironment, *EMT* epithelial-mesenchymal transition*, TP53* tumor protein 53, *BFT B. fragilis* toxin, *SCC* squamous cell carcinoma, *6-HAP* 6-N-hydroxyaminopurine.

A variety of microorganisms are present in tumor tissues [11], and intratumoral bacteria are more prevalent in these tissues than in normal tissues [9, 12, 13]. At the phylum level, *Firmicutes*, *Chordata*, *Bacteroidetes*, *Fusobacteria*, and *Planctomycetes* are more prevalent in tumor tissues [14]. Bacterial diversity increases with tumor growth and progression, which corresponds to the activation of characteristic cancer pathways [9]. Functionally specialized bacteria are preferentially enriched in certain types of cancer. For example, microorganisms that metabolize tobacco-derived compounds are enriched in lung cancer [9].

In addition to bacteria, the microbiome consists of viruses, fungi, and parasites [15]. Various oncogenic viruses (e,g., HPV and EBV) and flatworms (e.g., *Schistosoma haematobium*) are directly associated with human cancers [16]. Moreover, 98% of viral particles in malignancies are bacteriophages, whereas only 2% are eukaryotic viruses [17]. Viral metagenomic signatures have been identified in lung adenocarcinomas and in more than 100 tumor tissues [18]. Tumor-infiltrating viruses may affect host cell genetics, thereby facilitating oncogenesis [19].

Additionally, the presence of intratumoral fungi, mainly *Candida* spp. and *Aspergillus* spp., may cause tumor-associated inflammation, affect patient prognosis, and act as biomarkers [18]. For example, the presence of *Malassezia globosa* in breast tumors is associated with a low survival rate [20]. *Candida* and *Malassezia* are often observed in gastrointestinal, breast, and lung cancers [12]. Importantly, fungi often coexist with particular bacteria in tumors [12], indicating that cross-kingdom interactions may influence treatment responses.

When the involvement of the intratumoral microbiota in solid tumor immunotherapy is assessed, it is critical to evaluate both the bacteria and their metabolic environment. Bacterial compounds such as lipopolysaccharide (LPS) and peptidoglycan activate immunological pathways [21], whereas bacterial exosomes may suppress mechanisms of antitumor immunity by modification of the TME [22–25]. Similarly, microbial metabolites bind to receptors on cancer and immune cells and cause metabolic changes and immunological activation [26–29]. Short-chain fatty acids (SCFAs), key microbe-derived metabolites that accumulate in tumors, can alleviate protumorigenic inflammation caused by myeloid cells, thereby regulating cell proliferation and the TME phenotype [30]. Secondary metabolites, especially thiopeptides, exert anticancer effects by suppressing proteasomal degradation and FOXM1 signaling [31]. Metabolite profiles are specific to each cancer type. For example, *Bifidobacterium*-derived inosine improves anti-CTLA-4 treatment efficacy in colorectal cancer [28, 32], whereas in hepatocellular carcinoma, microbial metabolites such as fatty acids promote tumor progression via direct interactions with tumor cells [33].

## The intratumoral microbiota and TME of solid tumors

The TME is a complex ecosystem containing tumor cells, infiltrating immune cells, tumor-associated fibroblasts, stromal cells, extracellular matrix, and vascular endothelial cells [34]. The TME, especially the tumor immune microenvironment (TIME), affects how the body reacts to ICIs [34, 35]. Specific immune cells trigger anticancer immune responses. For example, high tumor CD8^+^ T-cell infiltration indicates a favorable response to ICIs [36]. Conversely, some immune cells inhibit the anticancer immune response. Cancer progression and treatment with different anticancer therapies continually affect the molecular and cellular activities of the TME and the influence of the TME on the anticancer immune response. The exploration of methods to alter the TME to increase the effectiveness of immunotherapy is a current research hotspot. Interestingly, several studies have indicated a link between the intratumoral microbiota, a key component of the TME, and the TIME and have indicated that certain microbes alter the TIME [32, 37–40]. Specific microbes can act as immune activators, inhibitors, or neutral factors in the TIME [30]. These results suggest that the intratumoral microbiota may increase immunotherapy efficacy by modifying the TME of solid tumors.

Many studies suggest the promising potential of the intratumoral microbiota in mediating solid tumor immunotherapy. Agents targeting the microbiome may reverse the immunosuppressive TME phenotype, highlighting a new option for improving the efficacy of ICIs and other treatments.

## The intratumoral microbiota affects different immune cells in the TME

The intratumoral microbiota can act as “living antigen reservoirs” and “natural immune adjuvants” that increase tumor antigenicity and immunogenicity. Microbial antigens may be presented by MHC class II-expressing dendritic cells (DCs) and prime T cells for activation in tumor-draining lymph nodes [41]. Microbes can also imitate tumor antigens by displaying bacterial peptides via HLA molecules, eliciting T-cell responses against malignancies [30]. Notably, such exogenous bacterial peptides increase immunogenicity compared with endogenous tumor antigens, and they can recruit immune cells and induce antitumor immunity by causing the release of tumor antigens and pathogen-associated molecular patterns (PAMPs) while promoting tumor-immunogenic cell death (ICD) [22, 23, 42]. In addition, the intratumoral microbiota may further regulate the activity, infiltration, and functional phenotype of tumor immune cells by secreting metabolites and producing cytokines. Understanding these complicated pathways allows us to obtain a better understanding of the critical functions that the intratumoral microbiota plays in TME remodeling.

### T cells

**Fig. 2:**
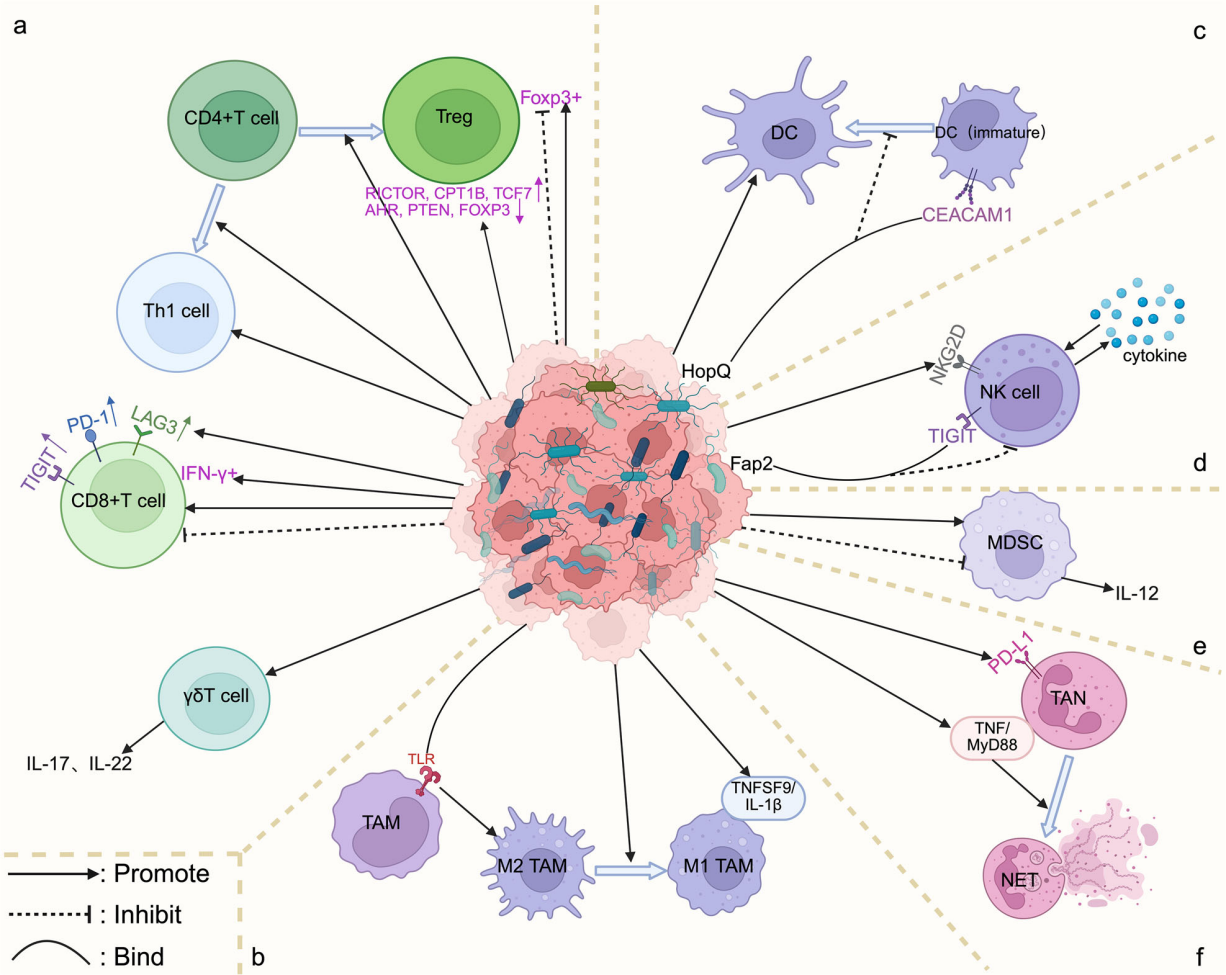
The intratumoral microbiota affects different immune cells. The intratumoral microbial community and its metabolic byproducts may modulate immune cell populations and functional states, potentially influencing cellular interconversion. **a** T cells Intratumoral microbiota-derived signals bidirectionally regulate T cell function: Some specific taxa promote Treg infiltration, while others suppress it. Metabolites can limit pro-tumor Treg responses and drive CD4⁺ T-cell differentiation toward either regulatory or Th1 phenotypes. CD8⁺ T-cell activity is either enhanced (via cytotoxicity and IFN-γ⁺ expansion) or suppressed (through exhaustion markers such as PD-1/LAG-3/TIGIT, or pathogen-mediated depletion). Specific taxa also activate γδ T cells. **b** Tumor-associated macrophages (TAMs) Intratumoral bacteria modulate TAM polarization via TLR signaling: by inducing M2 polarization through TLR4 and promoting M1 polarization via TLR2/9. Furthermore, pathogen (e.g., *F. nucleatum*) load also triggers TNFSF9/IL-1β–driven M1 polarization. **c** Dendritic cells (DCs) Microbiota enhances infiltration, whereas pathogens (e.g., *Neisseria*) impair maturation via HopQ-CEACAM1binding. **d** Natural killer (NK) cells Intratumoral *F. nucleatum* suppresses cytotoxicity via Fap2-TIGIT binding. Intratumoral viruses activate directly (e.g., NKG2D) or indirectly via cytokines. **e** Myeloid-derived suppressor cells (MDSCs) Fungal/bacterial components control recruitment: pathogens expand suppressive MDSCs; *L. monocytogenes* reduces abundance and enhances IL-12 immunocompetence. **f** Tumor-associated neutrophils (TANs) Dominant pathobionts drive PD-L1⁺ TAN accumulation. Metabolites (e.g., TMAO) induce NET formation via TNF/MyD88 activation. Created with BioRender.com.

T cells are a crucial part of adaptive immunity and include cytotoxic CD8^+^ T cells and CD4^+^ helper T cells [43]. They play a dual role in the TME: antitumor effector CD8^+^ T cells recognize and kill tumor cells, whereas immunosuppressive regulatory T cells (Tregs) inhibit immune cell functions and promote tumor immune escape [43]. Overactivation of γδ T cells may also promote tumor progression [44] (Fig. 2). The intratumoral microbiota and its metabolites influence T-cell recruitment, activation, functional differentiation, and depletion status [4, 30], reshaping the TME and influencing the antitumor immune response.

#### CD8^+^T cells

CD8^+^ T cells are important antitumor effector cells in the TME. They may destroy tumor cells and activate other immune cells by producing perforin, granzymes, and effector factors (e.g., IFN-γ) [45]. Research shows that particular intratumoral microorganisms or their metabolites have the capacity to alter these effector activities. *Streptococci*, for example, increase CD8^+^ T-cell infiltration while upregulating GZMB expression [46]. *Lactobacillus* Royce activates aryl hydrocarbon receptor (AHR), leading to the local expansion of IFN-γ^+^CD8^+^ T cells [47], whereas *Lachnoclostridium* increases the activity of cytotoxic CD8^+^ T cells [48] (Fig. 2). These results demonstrate the ability of certain intratumoral microbiota to favorably reinforce antitumor CD8^+^ T-cell responses.

However, intratumoral microbial control of CD8^+^ T cells is complicated and may lead to paradoxes. Sustained antigen exposure can cause CD8^+^ T cells to enter a state of T-cell exhaustion (Tex). This is characterized by a decline in effector functions (e.g., IFN-γ and GZMB secretion) and proliferative capacity, as well as upregulation of inhibitory receptors (e.g., PD-1, LAG3, and TIGIT) [49]. *Lactobacillus rohita* dramatically upregulates the expression levels of depletion-associated receptors on the surface of CD8^+^ T cells [47] (Fig. 2). Moreover, certain microorganisms and their metabolites can directly negatively regulate CD8^+^ T cells. Certain strains of *Escherichia coli* (*E. coli*) and *Fusobacterium nucleatum (Fn)* reduce the number of CD8^+^ T cells [23, 24, 32] (Fig. 2), whereas intratumoral *Methylbacilli* cause the dysfunction of tumor tissue-resident memory CD8^+^ T cells [50]. *Succinate* suppresses CD8^+^ T-cell infiltration by blocking the cGAS-interferon-β pathway [51] (Fig. 2). These findings suggests that the intratumoral microbiota can finely regulate the fate of key effector CD8^+^ T cells, as well as the complex mechanisms by which they may contribute to the immunosuppressive milieu, emphasizing the importance of understanding the specific context of their action (*e.g*., microbial species, abundance, and tumor type).

#### CD4^+^ T cells (including Tregs)

In the TME, CD4⁺ T cells enhance the function of CD8⁺ T cells and DCs by forming helper T cells (e.g., Th1), while their differentiated Tregs mediate immune tolerance by suppressing the immune response, forming the core of the bidirectional regulation of antitumor immunity [52]. Anti-PD-1/PD-L1 treatment suppresses the differentiation of Tregs while weakening their suppressive power [53]. Similar to this bidirectional modulation of Tregs by immunotherapy, the intratumoral microbiota has diverse modulatory effects on Tregs. *Stenotrophomonas* spp., *Selenomonas* spp. and *Cutibacterium acnes* promote an immunosuppressive milieu by increasing Treg infiltration [32, 54], whereas *Fn* may dramatically lower FoxP3⁺ Treg infiltration [55] (Fig. 2). Furthermore, the metabolite trimethylamine oxide (TMAO) limits the tumor-promoting Treg response by upregulating the expression of pro-effector genes (RICTOR, CPT1B, and TCF7) and downregulating the expression of suppressor-related genes (AHR, PTEN, and FOXP3) [56] (Fig. 2).

Furthermore, several intratumoral microbial metabolites influence CD4^+^ T-cell differentiation. Butyrate, an SCFA, helps differentiate CD4⁺ T cells into regulatory subpopulations and promotes effector responses such as Th1 differentiation [57] (Fig. 2). Inosine stimulates Th1 development and improves both antitumor immunity and the immunotherapy response by activating the T-cell-specific adenosine A2A receptor (A2AR) signaling pathway [28] (Fig. 2).

### Natural killer (NK) cells

Unlike antigen-specific killer T cells, NK cells are the primary cytotoxic cells of the natural immune system and the first line of defense against malignancies [58]. NK cells can directly release perforin and granzyme to lyse tumor cells [58]. However, the Fap2 protein secreted by intratumoral *Fn* can inhibit this killing effect by binding to the TIGIT receptor on the surface of NK cells, and xenograft studies have shown that TIGIT-neutralizing antibodies can reverse this inhibitory effect [59] (Fig. 2). In addition, NK cells can produce cytokines such as IFN-γ to trigger adaptive immune responses and mediate antibody-dependent cytotoxicity (ADCC) [58]. Notably, an increased abundance of *Bifidobacterium* enhances NK cell function via the metabolite hippurate, resulting in melanoma regression [60] (Fig. 4). Intratumoral viruses can also activate NK cells directly through activation receptors (e.g., NKG2D) or indirectly by producing cytokines that increase their activity [58] (Fig. 2).

### Tumor-associated macrophages (TAMs)

**Fig. 3:**
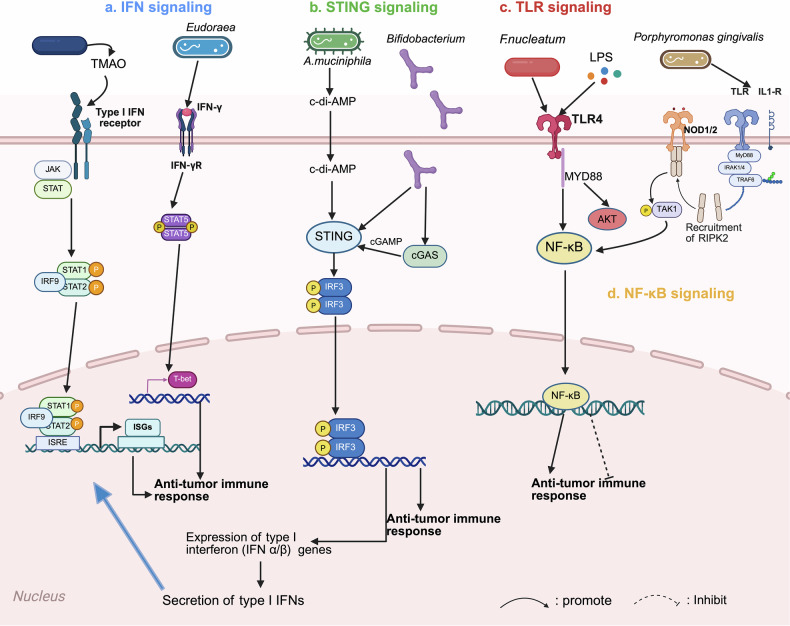
The intratumoral microbiota affects immunotherapy-related regulatory pathways in solid tumors. The intratumoral microbiota reshape the TME via complex interactions with major immune signaling pathways, such as IFN, STING, TLR, and NF-κB. **a** IFN signaling The microbial metabolite TMAO enhances antitumor immune responses through activation of the the type I interferon (IFN-γ) signaling, while *Eudoraea* in melanoma enhances antitumor immunity through the IFN-γ signaling. **b** STING signaling *A. muciniphil* synthesizes c-di-AMP to stimulate the STING/IRF3/type I interferon axis, thereby augmenting ICB responsiveness in melanoma; *Bifidobacterium* activates STING through cGAS recognition, thereby facilitating immunotherapy. **c** TLR signaling *F. nucleatum* activates AKT and NF-κB signaling by acting on TLR4; LPS similarly interacts with TLR4 receptors, amplifying NF-κB signal transduction and reinforcing antitumor therapeutic efficacy. **d** NF-κB signaling *Porphyromonas gingivalis* stimulates the IRAK/NF-κB cascade while concurrently elevating NOD1/2 signaling pathway, upregulates PD-1 in prostate cancer, and enhances antitumor immunity. Moreover, *F. nucleatum* can also cause immune rejection by activating NF-κB. Created with BioRender.com.

**Fig. 4:**
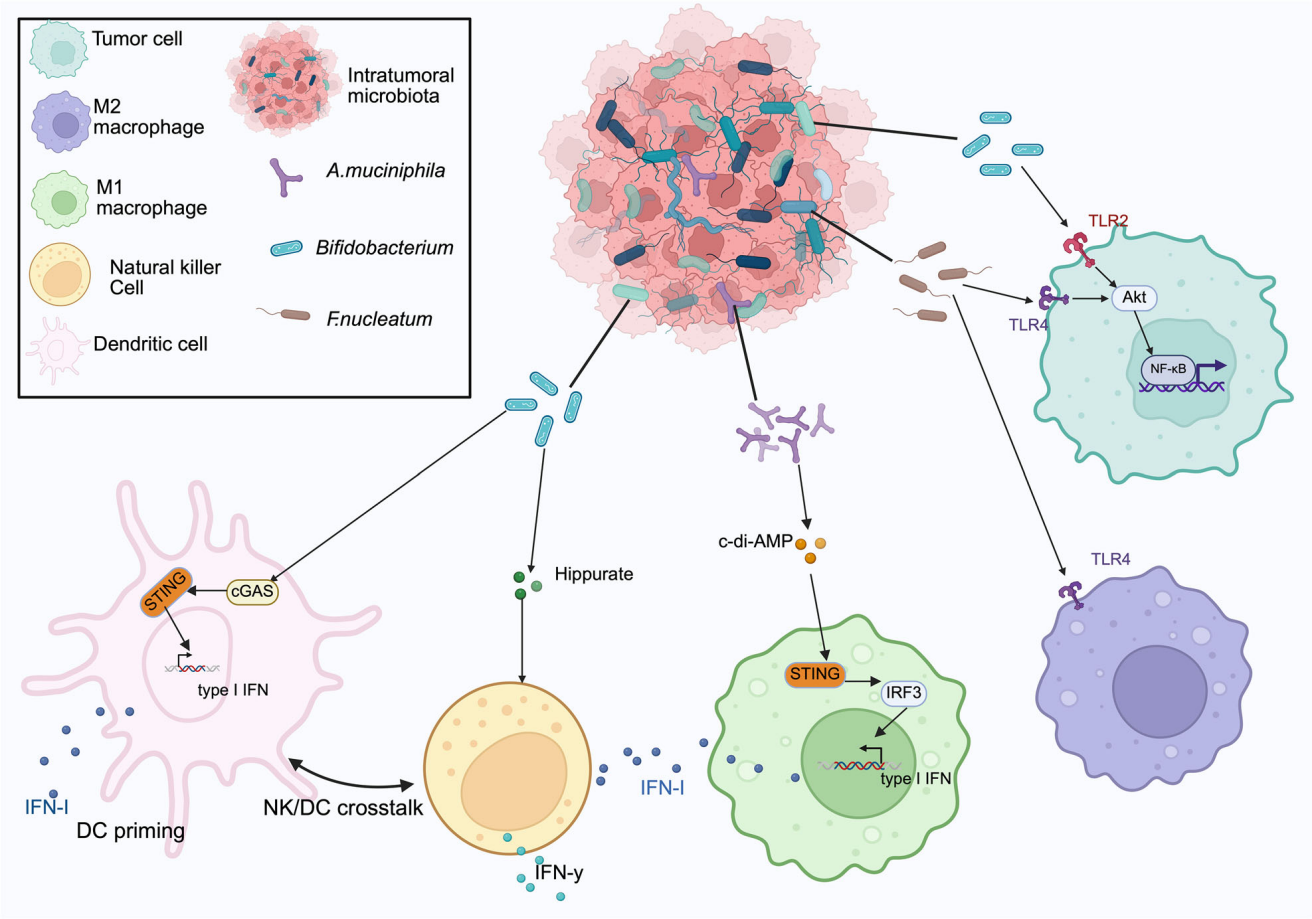
Microbiota-mediated modulation of the STING or TLR signaling pathways. Crosstalk between signaling pathways is a key mechanism underlying the immunoregulatory function of the intratumoral microbiota. *Bifidobacterium* activates the STING signaling pathway via bacterial DNA-induced cGAS recognition, initiating DC and promoting immunotherapeutic efficacy. Moreover, an increased abundance of *Bifidobacterium* enhances NK cell function via the metabolite hippurate, which leads to melanoma regression. Similarly, c-di-AMP produced by *A. muciniphila* activates the STING/IRF3/IFN-I signaling pathway, inducing antitumor macrophage polarization, promoting macrophage reprogramming and NK-DC crosstalk, and ultimately enhancing the efficacy of ICB in melanoma patients. *F. nucleatum* and *A. muciniphila*, binds to TLR4 and TLR2 on tumor cells, activating AKT and NF-κB signaling, while *F. nucleatum* promotes macrophage M2 polarization through a TLR4-dependent mechanism, which plays an immunosuppressive role. Created with BioRender.com.

Tumor-associated macrophages (TAMs) are functionally heterogeneous. The M1 type activates antitumor immunity by secreting inflammatory factors such as IL-12, whereas the M2 type promotes tumor progression by secreting immunosuppressive substances, including IL-10, making it a key regulatory hub in the TME [61]. Macrophages express pattern recognition receptors (PRRs) that can be recognized and bound by bacterial-derived molecules (e.g., LPS), resulting in significant immune cell migration and activation of the immune system to detect and eliminate tumor cells [62]. The Toll-like receptor (TLR) is the most extensively investigated PRR subtype [30]. Microbial activation of TLRs serves a dual function in the TIME [30]. Intratumoral bacteria may train TAMs via the TLR signaling pathway [63] (Fig. 2). *Fn*, for example, increases macrophage M2 polarization via a TLR4-dependent mechanism, causing immunosuppression [64] (Figs. 3, 4). However, microbial-derived D-lactic acid can promote macrophage polarization from the M2 phenotype to the M1 phenotype by interacting with TLR2 and/or TLR9, inhibiting the PI3K/Akt pathway and activating the NF-κB pathway [65] (Fig. 2). Combining TLR agonists with IFN-γ may increase the levels of the proinflammatory cytokines TNF-α, IL-12p40, and IL-12p70 while reducing IL-10 levels [66]. This creates an inflammatory microenvironment and promotes macrophage polarization toward the M1 phenotype [66, 67]. Therefore, precise regulation of the TLR pathway in combination with IFN-γ is critical for optimizing intratumoral microbe-mediated remodeling of TAM function.

In addition to the TLR pathway, the intratumoral microbiota controls TAM activity via a variety of nonclassical mechanisms. A high *Fn* load increases TNFSF9/IL-1β signaling, leading to M1 macrophage polarization [68] (Fig. 2). *Akk*-produced cyclic di-AMP (c-di-AMP) stimulates STING/IRF3/IFN-I signaling, polarizing macrophages toward the M1 phenotype and improving NK-DC interactions [69] (Figs. 3, 4). TMAO also enhances the immunostimulatory function of TAMs through the IFN-I pathway [56] (Fig. 3). In contrast, tryptophan derivatives activate AHRs, inhibiting the anticancer action of TAMs [70]. This research shows that intratumoral microorganisms exert complicated and multidirectional regulatory effects on the TAM phenotype via metabolites, bacterial signaling molecules, and multipathway crosstalk.

### Other myeloid cells

Intratumoral microorganisms may modify immune responses by affecting other myeloid cells in the TME. DCs are the main antigen-presenting cells in the TME and bridge innate and adaptive immunity by taking up, processing, and presenting tumor antigens to T cells [52]. DC function is often affected by microenvironmental inhibition, which in turn dynamically modulates the activation or tolerance status of the antitumor immune response [52]. Notably, the intratumoral microbiota (such as *Stenomonas* spp., *Selenomonas* spp., and HPV) may stimulate DC infiltration in tumor tissues [32] (Fig. 2). *Bifidobacterium bifidum* improves DC function in mice by activating CD8^+^ T cells [71] (Fig. 4). The oral ingestion of *Lactobacillus rhamnosus GG* may increase the number of DCs and attract CD8^+^ T lymphocytes via IFN signaling [21]. However, intratumoral pathogenic *Neisseria* strains impair normal immature DC maturation by the binding of its HopQ protein to CEACAM1 [72] (Fig. 2). All the information presented above suggests that the intratumoral microbiota exerts multiple regulatory effects on DCs.

Myeloid-derived suppressor cells (MDSCs) are present in the blood of patients with cancer and may be attracted to cytokines, travelling to the tumor site via the peripheral circulation [21, 73]. MDSCs act as a significant immunosuppressive barrier to tumor growth by suppressing T-cell activity and facilitating immunological escape in the TME [73]. High levels of MDSC infiltration in tumor tissues might indicate a poor prognosis and treatment resistance [74]. *Candida tropicalis* mediates immunosuppression by increasing MDSC recruitment [75], whereas *Listeria monocytogenes* decreases MDSC numbers and stimulates IL-12 production by the remaining MDSCs, resulting in an immunocompetent phenotype [21] (Fig. 2).

Tumor-associated neutrophils (TANs) have two functional phenotypes in the TME. The antitumor N1 phenotype enhances immune surveillance through the release of CXCL10 and reactive oxygen species (ROS). In contrast, the TGF-β-induced protumor N2 phenotype secretes factors such as neutrophil elastase (NE), which drive angiogenesis, matrix remodeling, and the formation of neutrophil extracellular trapping networks (NETs), promoting metastasis [76, 77]. *Fn* activates the IL-17/NF-κB/RelB signaling pathway in tumor cells, leading to considerable TAN recruitment [78]. Furthermore, intratumoral bacteria can indirectly stimulate neutrophils to overexpress NE and matrix metalloproteinase-9 (MMP-9), resulting in polymorphonuclear leukocyte (PMN) activation and NET formation as a physical barrier between tumor cells and NK cells, mediating innate immune escape [77]. Furthermore, the microbiome-derived metabolite TMAO promotes NET formation via TNF/MyD88 activation while suppressing PPARG-mediated anti-inflammatory responses [56] (Fig. 2).

## The intratumoral microbiota affects immunosuppressive checkpoint (e.g., PD-1/PD-L1) expression in the TME

The development of immune checkpoint inhibitors has resulted in significant breakthroughs in tumor treatment, with the most often employed immune checkpoints being PD-1, TIM-3, CTLA-4, LAG-3, CEACAM1, and TIGIT [30]. Among these, PD-1 (programmed death receptor-1) and its ligand PD-L1 form a key pathway that promotes tumor immune escape via T-cell depletion and functional inhibition [79]. PD-1 expression in the TME is regulated by multiple mechanisms. *Aspergillus sydowii* accumulation in lung adenocarcinoma (LUAD) increases PD-1 expression on CD8⁺ T cells [74], and the gram-negative bacterial LPS upregulates PD-1 through the NF-κB/IL-6 pathway [80] (Fig. 3). *Helicobacter pylori* (*H. pylori*) upregulates PD-L1 in gastric cancer cells via CagA-dependent SHH signaling [81, 82], and *Porphyromonas gingivalis* upregulates PD-L1 in prostate cancer via the IRAK/NF-κB pathway [83] (Fig. 3). In colorectal cancer, the *Fn-*Dps protein increases PD-L1 expression through ATF3-dependent promoter activation [84].

Interestingly, specific intratumoral microbes promote AhR expression in pancreatic cancer, whereas AhR deficiency increases MHC-II, CD40, and PD-L1 expression in the TME [70]. However, microbial clearance with antibiotics in a mouse model of pancreatic cancer greatly increases T-cell activation and PD-1 expression [63], indicating the presence of complicated interactions with the intratumoral microbiota. The intricate involvement of the intratumoral microbiota is also associated with the type of solid tumor. Butyrate, a microbial metabolite, inhibits tumor cell growth in gastric cancer by suppressing PD-L1 expression as well as NF-κB and STAT3 expression [85]. In contrast, in melanoma, butyrate inhibits histone deacetylase (HDAC) in a GPR-independent manner, upregulates the expression of PD-1/PD-L1, and inhibits the death of CD4^+^ T cells [86–88]. In addition, in a gastric cancer mouse model, *Fn* invasion caused PD-L1⁺ TAN aggregation by activating the IL-17/NF-κB pathway, inhibiting CD8⁺ T-cell function, and resulting in immunological escape [78] (Fig. 2). However, overall, the high-*Fn*-load group showed more intratumoral PD-L1⁺TANs and a better response to anti-PD-L1 treatment. Depleting neutrophils reduces the effectiveness of anti-PD-L1 therapy [78]. The discovery that the intratumoral microbiota alters solid tumor immunotherapy efficacy by changing the expression of PD-L1 immunosuppressive checkpoints necessitates a comprehensive approach.

## The intratumoral microbiota modulates immunotherapy efficacy across solid tumors

Emerging evidence highlights the tumor type-specific roles of the intratumoral microbiota in shaping immunotherapy outcomes via diverse immunomodulatory pathways.

### Melanoma

**Fig. 5:**
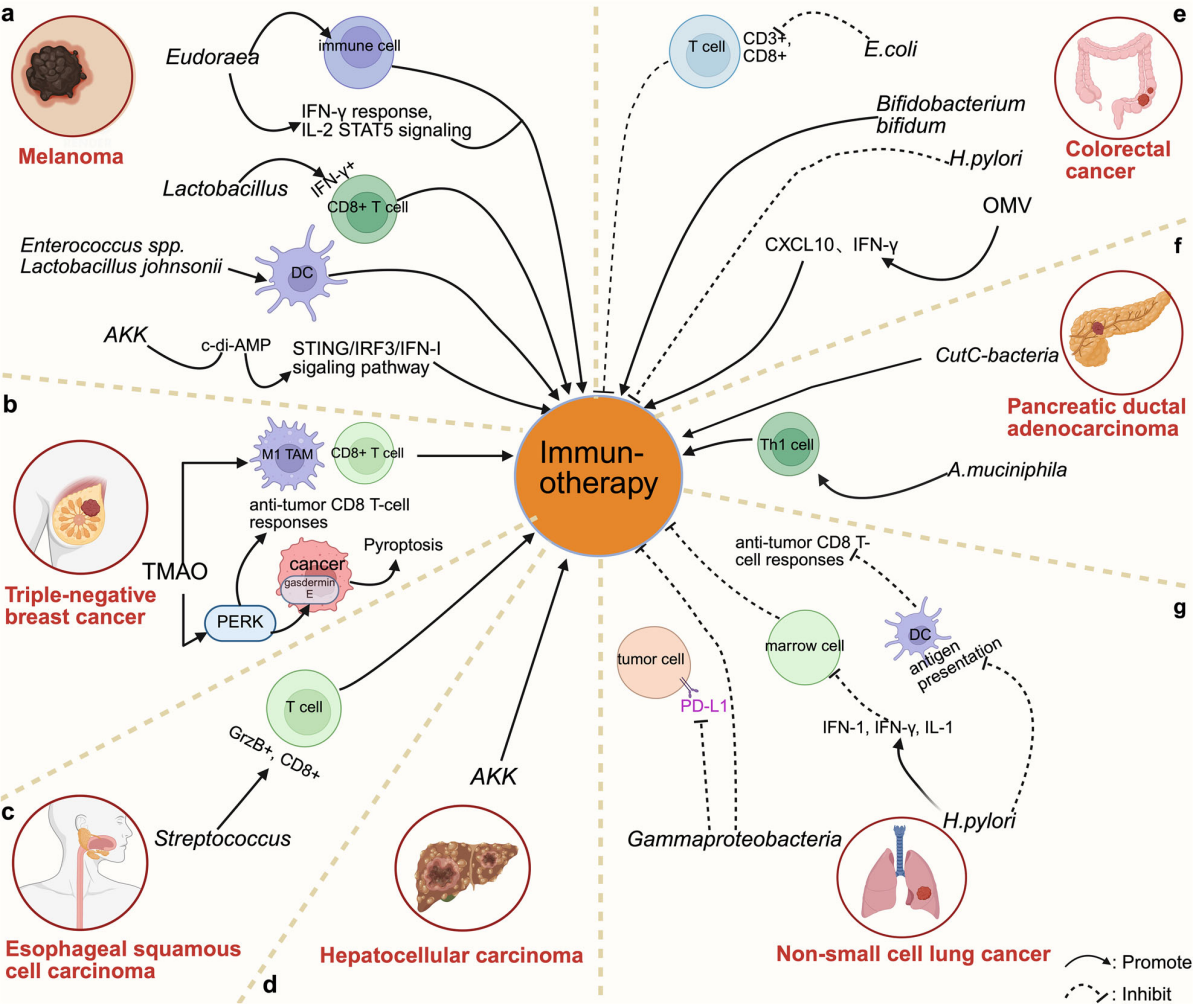
The intratumoral microbiota affects solid tumor immunotherapy. **a** Melanoma The intratumoral microbiota enhances melanoma immunotherapy through multiple mechanisms: *Eudoraea* increases active immune cells and enhances IFN-γ and IL-2/STAT5 signaling; *Lactobacillus* enhances IFN-γ^+^CD8^+^ T cells; *Enterococcus spp*. and *Lactobacillus johnsonii* stimulate DC maturation to initiate T-cell-mediated immunity; and *A. muciniphila* (*AKK*) synthesizes c-di-AMP to activate the STING/IRF3/IFN-I axis. **b** Triple-negative breast cancer (TNBC) The microbial metabolite TMAO activates PERK signaling, induces gasdermin E-dependent tumor cell pyroptosis, and promotes M1 macrophage polarization coupled with CD8^+^ T-cell activation, collectively enhancing anti-PD-1 responsiveness. **c** Esophageal squamous cell carcinoma (ESCC) *Streptococcus* enrichment enhances GrzB^+^CD8^+^ T-cell infiltration and potentiates anti-PD-1 efficacy. **d** Hepatocellular carcinoma (HCC) Reduced levels of *A. muciniphila* (*AKK*) are associated with poor immunotherapy efficacy. **e** Colorectal cancer (CRC) *E. coli* suppresses immunotherapy by inhibiting CD3^+^CD8^+^ T cells; *H. pylori* also inhibits immunotherapy efficacy; *Bifidobacterium bifidum* enhances anti-CD47 functionality; and OMVs may induce CXCL10 and IFN-γ to promote antitumor immunity. **f** Pancreatic ductal adenocarcinoma (PDAC) *A. muciniphila* enhances the Th1 immune response and thus enhances antitumor immunity; *CutC-bacteria* also enhance the effectiveness of ICIs. **g** Non-small cell lung cancer (NSCLC) *H. pylori* impairs DC-mediated antigen cross-presentation to suppresses antitumor CD8^+^ T-cell responses and inhibits bone marrow cells via IFN-I, IFN-γ, and IL-1 to reduce immunotherapy efficacy in NSCLC. *Gammaproteobacteria* enrichment is associated with decreased PD-L1 expression and poor outcomes. Created with BioRender.com.

The diversity of the melanoma microbiome has a significant effect on immune checkpoint blockade (ICB) effectiveness. In response to anti-PD-1/CTLA-4 treatment, patients exhibit enrichment of *Eudoraea* and *Desulfonatronospira*, which enhance T-cell activation via interferon-γ/STAT5 pathway upregulation and increased immune cell infiltration [89] (Figs. 3, 5). *AKK* binds to TLR2 on tumor cells, thereby activating AKT/NF-κB and improving the efficacy of IL-2 therapy in patients with melanoma [90] (Fig. 4). Conversely, *H.pylori* infection in B16-OVA mice inhibits innate immunity, thus increasing inflammatory cytokine levels and blunting vaccine-induced responses [91]. Strikingly, *Enterococcus* spp. and *Lactobacillus johnsonii* counteract this suppression by activating DCs, thereby restoring anti-PD-1/CTLA-4 efficacy [92] (Fig. 5).

### Colorectal cancer (CRC)

CRC-associated bacteria, such as colistin-producing *E.coli* induce DNA damage via ROS and UshA nuclease activity, which may increase the neoantigen load to increase sensitivity to immunotherapy [93]. Furthermore, similar to *Fn*, *E. coli* drives resistance to immunotherapy via dual mechanisms—by decreasing the number of tumor-infiltrating CD3^+^CD8^+^ T cells and by upregulating PD-L1 expression [32, 46] (Figs. 5, 7). *H. pylori* infection increases resistance to CTLA4 blockade in AOM/DSS-induced and MC38 CRC models [82]. In contrast, *Bifidobacterium bifidum* activates STING by recognizing bacterial DNA with cGAS, thereby increasing anti-CD47 efficacy [94] (Fig. 3). *Lactobacillus gallinarum* improves anti-PD-1 responses by reducing Foxp3^+^CD25^+^ Treg infiltration while enhancing CD8^+^ T-cell effector functions [95], whereas bacterial OMVs can increase CXCL10/IFN-γ production to synergize with ICB [96] (Fig. 5).

### Breast cancer (BC)

*Clostridium difficile*-associated TMAO promotes PERK-dependent tumor cell pyroptosis in triple-negative breast cancer (TNBC), thereby increasing M1 macrophage and CD8^+^ T-cell infiltration to sensitize tumors to anti-PD-1 therapy [97] (Fig. 5). Parallel studies revealed that reducing intratumoral *Fn* expression remodels the immunosuppressive TME, thereby potentially overcoming PD-1 resistance [98] (Fig. 7).

### Pancreatic ductal adenocarcinoma (PDAC)

**Fig. 6:**
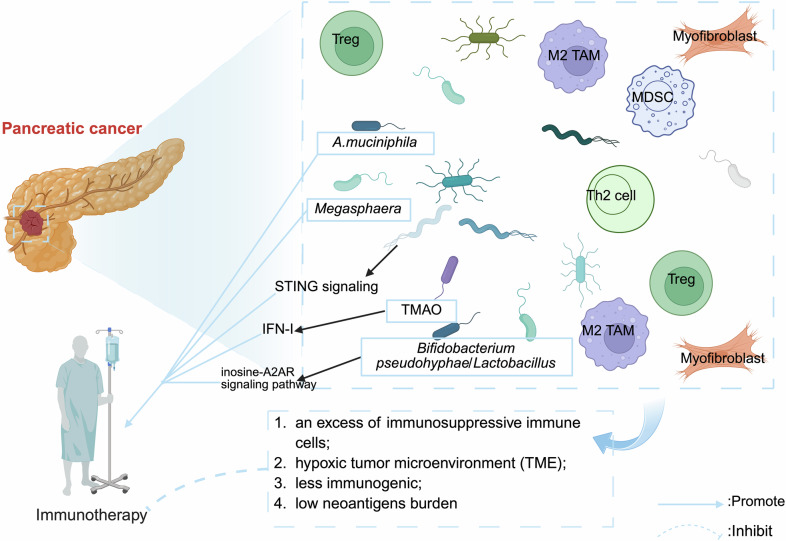
Impact of the intratumoral microbiota on immunotherapy efficacy in patients with PDAC. Despite the success of immune checkpoint inhibitors (ICIs) in other solid tumors, PDAC remains refractory due to its unique immunosuppressive microenvironment: 1. An excess of immunosuppressive immune cells (e.g. M2-like TAMs, MDSCs, Tregs, and Th2 cells) creates a “cold” microenvironment; 2. Collapsed tumor vasculature and pancreatic ducts result in a hypoxic tumor microenvironment (TME) and limit drug delivery; 3. Loss of MHC-I expression contributes to diminished immunogenicity; 4. A low level of neoantigen expression, resulting from a low tumor mutational burden (TMB), leads to a poor response to immunotherapy. The intratumoral microbiota can reverse this through mechanisms including: *Megasphaera* treatment enhances tumor growth suppression; Microbiota-derived STING agonists make the TME more conducive to the immune response; The microbiome-derived metabolite TMAO bolsters the efficacy of ICIs in PDAC by regulating the IFN-I pathway; *A. muciniphila* improves the effectiveness of anti-PD-L1 therapy; *B. pseudomallei /Lactobacillus/Johnson*’s inosine-A2AR signaling pathway facilitates ICI treatment. Created with BioRender.com.

The limited efficacy of immunotherapy in PDAC patients is due to factors such as its unique TME, which lacks CD8^+^ T cells but possesses CD4^+^ T cells, specifically Th2 cells and Tregs [99–101] (Fig. 6). The TME of PDAC is characterized by an excess of immunosuppressive cells, thus creating an immunosuppressive TME [99–101] (Fig. 6). Additionally, the absence of MHC-I expression on the surface of PDAC cells renders them less immunogenic [99], thereby leading to a low tumor mutation rate and a poor response to immunotherapy (Fig. 6). The long-term survival of PDAC patients is associated with increased CD3^+^CD8^+^ T-cell and GzmB^+^ cell counts [10] (Fig. 6). Recent research has indicated that the intratumoral microbiota (or its metabolites) may improve the effectiveness of PDAC immunotherapy [28, 30, 56, 69, 102–105] (Fig. 6).

### Esophageal squamous cell carcinoma (ESCC)

*Streptococcus*-dominated tumors respond better to neoadjuvant anti-PD-1 chemotherapeutic combinations, and this improved response is associated with increased GrzB^+^CD8^+^ T-cell infiltration [46] (Fig. 5).

### Hepatocellular carcinoma (HCC)

A reduced abundance of *AKK* is associated with faster HCC development and decreased immunotherapy efficacy [106] (Fig. 5), which contradicts its protective effect in melanoma.

### Non-small cell lung cancer (NSCLC)

*Gammaproteobacteria*-enriched tumors exhibit low PD-L1 expression, which impairs poor checkpoint inhibitor responses [107]. Furthermore, *H. pylori* further impairs DC-mediated CD8^+^ T-cell activation via IFN-I/IL-1 pathway suppression, thereby worsening anti-PD-1 resistance in NSCLC [91] (Fig. 5).

### Cross-tumor microbial dynamics

**Fig. 7:**
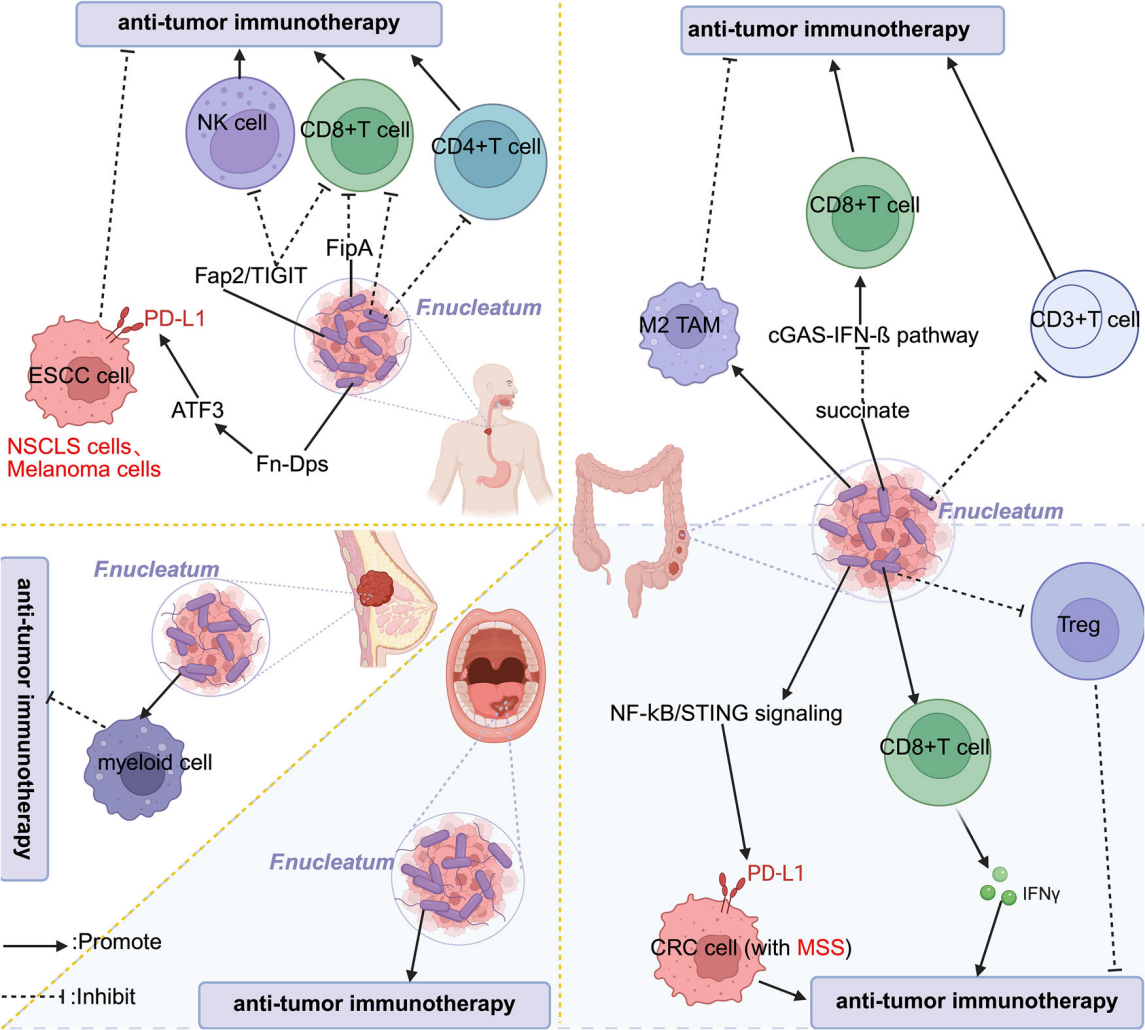
Role of *F. nucleatum* in immunotherapy efficacy in solid tumors. The intratumoral microbiota, such as *Fusobacterium nucleatum* (*Fn*), demonstrates dual pro- and anti-tumorigenic roles through immunomodulatory mechanisms across tumor types. (1) Pro-Tumorigenic Effects of *Fn* Across Tumor Types. In colorectal cancer (CRC), *Fn* reduces FoxP3^+^ Tregs and increases M2 macrophages, while its associated succinate suppresses CD8^+^ T-cell infiltration via inhibition of the cGAS–IFN-β pathway. In esophageal squamous cell carcinoma (ESCC), non-small cell lung cancer (NSCLC), and melanoma, *Fn*-Dps upregulates PD-L1 through ATF3-dependent activation, leading to T-cell suppression. Notably, in ESCC, this is mediated by FipA-induced inactivation and Fap2/TIGIT binding, which inhibits NK/T cell killing and αPD-L1-mediated CD4^+^/CD8^+^ T-cell proliferation. In breast cancer (BC), *Fn* recruits immunosuppressive myeloid cells, contributing to an immunosuppressive tumor microenvironment (TME). (2) Anti-Tumorigenic Effects of *Fn* in Specific Contexts. In CRC, elevated *Fn* levels correlate with improved response to PD-1 inhibition, mediated through NF-κB/STING-mediated PD-L1 elevation and IFN-γ^+^CD8^+^ TIL proliferation. In oral squamous cell carcinoma (OSCC), *Fn* enrichment is associated with a better patient prognosis. Created with BioRender.com.

Similar to *Fn*, the effects of the intratumoral microbiota on the results of immunotherapy can vary by species and tumor type. *Fn* reduces immunological efficacy in ESCC by upregulating PD-L1 and suppressing T cells [84], but *Fn* improves the PD-1 response in OSCC by activating the STING/NF-κB pathway [108] (Fig. 7). *Fn* also exerts both pro- and anticancer effects on CRC through these pathways [51, 55] (Fig. 7). The dual effects of *Fn* within tumors are dependent on the tumor type and microenvironment signaling pathways [37, 51, 55, 84, 108–112]. Moreover, *AKK* improves ICB effectiveness in melanoma and PDAC but protects against HCC [103, 106], whereas *H.pylori* generally decreases responses in both gastrointestinal and pulmonary cancers [91]. Therapeutic predictions are further complicated by strain-level functional heterogeneity, such as the heterogeneity observed between *E. coli* that do and do not produce colistin. Clarification of the mechanism of microbial–immune interplay and spatial mapping of tumor-resident populations will be essential for creating microbiota-guided combination therapies.

## Influence of MSI status on the intratumoral microbiota and immunotherapy efficacy in solid tumors

Emerging evidence also suggests that microsatellite instability (MSI), an inherent tumor characteristic, together with the intratumoral microbiota, affects treatment outcomes [4]. For example, *Fn* suppresses T-cell infiltration in MSI-H-type CRC but enhances TIL recruitment in MSS-type CRC [113]. In addition, MSI (a crucial cancer marker) is associated with increased tumor immune and mutational loads [114]. Tumors with an MSI-high (MSI-H) phenotype typically exhibit greater mutational loads and better responses to anti-PD-1 therapies [114]. Additionally, different solid tumors with the same MSI status exhibit different immune responses, with stronger immune responses to dMMR/MSI observed in CRC than in EC [115].

We also observed that the intratumoral microbial composition and abundance differ among different tumor types with the same MSI status. For example, CRC patients with the same MSI-H status present a greater variety of oral genera. Additionally, the composition and abundance of the intratumoral microbiota differ for the same tumor types in different MSI states. For example, *Mycobacterium* spp. and *Prevotella* spp. are more prevalent in MSI-H CRC tumors on average. Microbes enriched in MSI-H tumors are also associated with greater expression levels of immune-related genes, such as CD3E and CD8E, which have favorable prognostic value [116].

Future research should focus on the use of spatial multiomics to resolve MSI-microbiome-immune circuits across tumor types and the functional validation of microbial consortia in modifying MSI-driven therapeutic vulnerabilities.

## Intratumoral fungi and their effects on tumor growth

Although discussions of intratumoral microbes tend to focus more on intratumoral bacteria, emerging evidence demonstrates that fungi play a significant role in the immunological response of the host. Specific fungi within tumors may directly drive solid tumor development by targeting immunological or metabolic pathways. These fungi may maintain chronic inflammation, promote the formation of an immunosuppressive TME, and activate oncogenic signaling pathways such as the EGFR pathway [74, 117, 118]. The targeting of fungal infections or related inflammatory pathways may be a novel strategy for solid tumor prevention and treatment.

Intratumoral fungi exert both protumor and antitumor effects by modulating the TME in multiple dimensions, and their functional heterogeneity varies greatly depending on the tumor type and fungal species. *Candida albicans* triggers the dectin-1-mediated Src-Syk-CARD9 pathway, which causes PDAC cells to release IL-33. Moreover, *Malassezia globosa* activates the MBL-complement C3 pathway. Both types of fungi recruit Th2 and ILC2 cells. Experimental results demonstrate that IL-33 inhibition or antifungal therapy may reduce the tumor burden and prolong survival time in mice [117]. In contrast, *A. sydowii* in LUAD affects the β-glucan/dectin-1/CARD9 axis by suppressing cytotoxic T cells and promoting the accumulation of PD-1^+^ CD8^+^ T cells, thus promoting LUAD progression [74].

The contradictory characteristics of fungal function are particularly noticeable in CRC. *Candida albicans* stimulates the IL-7/IL-22-ILC3 axis via macrophage HIF-1-dependent glycolysis, thereby driving immunosuppression and MDSC accumulation [119]. The CARD9 protein activates the NF-κB pathway via surface receptors, which prevents colorectal carcinogenesis by reducing fungal-mediated MDSC proliferation [119]. Moreover, tropical *Candida* species can enhance the immunosuppressive function and intratumoral accumulation of bone marrow-derived suppressor cells via PKM2-dependent glycolysis, thereby increasing colorectal carcinogenesis. Interestingly, fungal invasion in CRC modifies the immunological microenvironment and enhances CD8^+^ T-cell performance by activating the dectin-1/Syk/IL-18 axis in myeloid cells. The myeloid cell SYK-CARD9 signaling axis may also prevent colitis-associated CRC by activating inflammasomes via commensal gut fungi such as *Candida*. In MSS CRC, fungal components that promote IL-17A activation, such as HKCA, can render tumors more susceptible to PD-1 inhibitors [120]. Conversely, antifungal pharmaceutical treatments can aggravate CRC, suggesting that intratumoral fungi may generate antitumor immunity under specific circumstances.

In addition, *Candida albicans*, which is involved primarily in CRC, may stimulate OSCC metabolism and carcinogenic signaling [12], thus indicating that the same fungus may promote cancer development in various tumor types. Furthermore, the diversity of fungal communities is significantly associated with tumor features such as MSI [121]. Moreover, the number of fungi within tumors and the enrichment of *Aspergillus* and mushroom fungi are greater in smokers than in nonsmokers, suggesting that environmental exposure may affect fungal composition by influencing local metabolism or immunological status [12]. Further studies are needed on the diversity of fungi and their mechanisms within tumors.

## Applications of the intratumoral microbiota in mediating solid cancer immunotherapy

According to recent research, three key factors are involved in a successful antitumor immune response: an antigen, an adjuvant, and a favorable immune microenvironment. The intratumoral microbiota can induce immunogenicity, facilitate localized payload delivery, and target tumors, improving the efficacy of precision therapy [122].

### Activation of antitumor immunity via bacterial agents

**Fig. 8:**
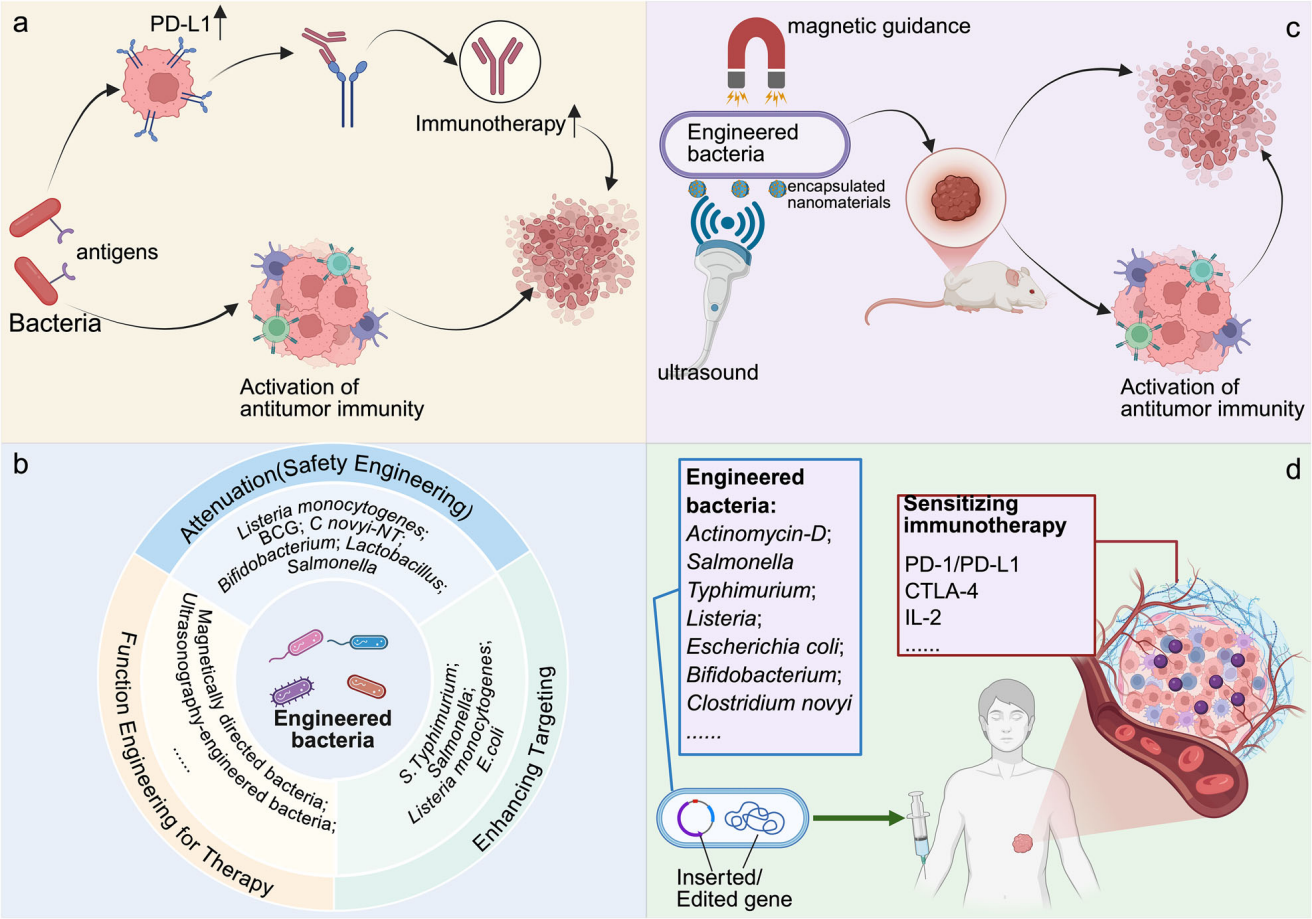
Clinical translation strategies for tumor-inhabiting microbiota-based therapeutics. **a** Immunotherapeutic sensitization. Intratumoral bacteria can be used as an antigenic source to improve the efficacy of immunotherapy by increasing PD-L1 expression in tumor cells and activating the antitumor immune response, thus effectively killing tumor cells. **b** Engineering bacteria. By modifying the natural intratumoral microbiota for safety and toxicity reduction, enhancing targeting and specific functions (e.g., drug delivery), therapeutic engineered bacteria can be constructed. **c** Targeted drug delivery. Engineered bacteria capable of magnetic or ultrasonic targeting may be utilized as targeted carriers to deliver nanodrugs to tumor locations, activate antitumor immunity, and kill tumor cells. **d** Clinical translation progress. Engineered bacteria developed on the basis of the above strategies are undergoing investigation in clinical trials for sensitizing solid tumors to immunotherapy (e.g., therapies targeting PD-1/PD-L1, CTLA-4, and IL-2). Created with BioRender.com.

This specific approach involves the use of either live or dead bacteria to activate antitumor immune responses via specific antigens. Examples of products based on this concept include the *Bacillus Calmette-Guérin* (BCG) vaccine for bladder cancer and the attenuated *Listeria monocytogenes* vaccines [5] (Fig. 8).

Bacterial colonization flourishes in hypoxic TME niches. This tropism allows for targeted immune activation. For example, in a phase I study (NCT01924689), intratumoral *C. novyi-NT* injections decreased the tumor burden and increased tumor-specific T-cell responses in resistant solid tumors [123] (Fig. 8). Similarly, the oral administration of *Bifidobacterium bifidum* combined with an anti-PD-L1 agent almost completely suppressed tumor development in a preclinical mouse study, suggesting improvement in microbiota-driven immunotherapy [124] (Fig. 8). Moreover, checkpoint inhibitor-producing bacteria may be directly targeted within tumor tissues to facilitate the binding and neutralization of T-cell suppressor sites expressed by tumor cells [125].

Engineered microbes improve targeting effects [126]. In a phase I/IIa experiment, modified *Lactobacillus casei* GLBL101c expressing the HPV16 E7 antigen decreased the severity of cervical neoplasia in 70% of patients by triggering E7-specific Th1 responses [127, 128] (Fig. 8). *Salmonella strains* carrying the asd gene can elicit tumor regression in bladder, colon, and melanoma mouse models [129] (Fig. 8). Moreover, *Coxsackievirus* A21 paired with pembrolizumab regulated PD-L1^+^ tumor cells in a phase Ib trial [130] (Fig. 8), whereas the combination of TG4010 (a modified vaccinia virus) with PD-1/PD-L1 inhibitors improved outcomes in advanced NSCLC patients in phase III research [131].

### Engineered bacteria as therapeutic vectors

Bacteria can function as low-toxicity delivery systems for tumor-targeted payloads [125]. In over 80% of glioblastoma multiforme (GBM) models, *Salmonella typhimurium* VNP20009 modified with SLIN nano-capsules extends survival beyond 90 days [132] (Fig. 8). *Salmonella*-mediated β-galactosidase administration results in robust immune responses [133], whereas *attenuated Listeria monocytogenes (Lm*^*at*^*-LLO)* is effective in melanoma models as a drug delivery platform [134] (Fig. 8). Furthermore, *E. coli* modified to emit nitric oxide causes tumor regression, thereby demonstrating bacterial adaptability in drug delivery [135] (Fig. 8).

### Integration of the microbiota with imaging-guided therapy

When combined with bacteria-based sensors and networks, engineered bacteria may offer helpful information regarding tumor existence, burden, and microenvironmental conditions [122]. Magnetically directed bacteria can target hypoxic tumors. Moreover, the noninvasive and focusable features of ultrasound make it a perfect tool for accurately visualizing and controlling modified microbes (Fig. 8). Ultrasonography-engineered bacteria known as Ec@DIG-GVs enable dual imaging and doxorubicin release and synergize with IFN-γ to activate antitumor T cells in the acidic TME [136] (Fig. 8).

These systems demonstrate the convergence of diagnostic and therapeutic techniques [5] (Fig. 8). Based on these research directions, several drugs are now under investigation in clinical trials, as detailed in Table 3.

**Table 3:** Clinical trials of antitumor bacterial therapies (https://beta.clinicaltrials.gov/.given by the US National Library of Medicine).

Microbial species	Medicines	Study design	Clinical trial phase	Identifier
*Actinomycin-D*		*Actinomycin-D* with Toripalimab as First-Line Therapy for GTN with FIGO Score 5–6 (TA56)		NCT06028672
		*Actinomycin-D* with Toripalimab as the first-line treatment for GTN with FIGO score 7 (TA7)		NCT06020755
*Salmonella Typhimurium*	VNP20009 [173]	Treatment of Patients with Terminal Solid Tumors Using VNP20009	Phase I	NCT00006254
		The Microbiome as a Biomarker in Locoregionally Advanced OSCC		NCT00004216 NCT00004988
	SGN1	Investigation of SGN1 Injections Intratumorally in Patients With Terminal Solid Tumors	Phase I/IIa	NCT05103345
		SGN1 in Patients With Terminal Solid Tumors	Phase I	NCT05038150
	SalpIL2 [174]	*Salmonella Typhimurium* in unresectable hepatic spread expresses IL-2	Phase I	NCT01099631
	VXM01 [173]	Phase I Pilot Study of VXM01 in Operable Glioblastoma Recurrence Patients	Phase I/II	NCT02718443
				NCT02718430, NCT01486329
	YB1 [175]		Pre-clinical	
multiple bacteria	MBV [176]	Anti-anxiety Biotics for Breast Cancer Survivors	Phase I	NCT00623831
*Listeria*	JNJ-64041809 [174]	Evaluating the Immunotherapy Profile of JNJ-64041809: A Double-deleted, Live-Attenuated *Listeria*-Based Therapeutic in Metastatic Castration-Resistant Prostate Cancer Patients	Phase I	NCT02625857
	JNJ-64041757 [174]	Immunotherapy Efficacy and Reactivity Profile of JNJ-64041757, a Genetically Attenuated Double-deleted *Listeria*-Based Therapy, in Patients with NSCC	Phase I	NCT02592967
	pLADD [174]	Clinical Evaluation of Tailored Immune-Targeted Therapy in Adult Patients with Metastatic CRC	Phase I	NCT03189030
	ADU-623 [174]	Immune Modulation and Safety Profiling of ADU-623	Phase I	NCT01967758
				NCT03006302
	CRS-207 [143, 174, 177]	Nivolumab, Ipilimumab, and CRS-207 With Optional CY/GVAX in Metastatic PDAC		NCT03190265
				NCT02243371, NCT02004262, NCT05014776
	ADXS11-001 [178]	ADXS 11-001 Administration Before Robotic Resection of HPV^+^ Oropharyngeal Cancer	Phase II	NCT02002182
				NCT02399813, NCT02853604, NCT02531854,
	ADXS31-164	ADXS31-164 in HER2-Driven Solid Tumors: A Phase 1b Dose Escalation and Safety Analysis	Phase I/II	NCT02386501
	ADXS-503	Phase 1 Clinical Trial Evaluating ADXS-503 Alone or in Combination with Pembrolizumab for Metastatic NSCLC	Phase I/II	NCT03847519
	ADXS-NEO	A Study of ADXS-NEO Expressing Personalized Tumor Antigens	Phase I	NCT03265080
	ADXS31-142	Personalized ADXS31-142 Regimens With/Without Pembrolizumab (MK-3475) in Prostate Cancer patients	Phase I/II	NCT02325557
*Escherichia coli*	SYNB1891 [179]		Phase I	NCT04167137
*Bifidobacterium longum*	APS001F [174]	APS001F with Maltose and Flucytosine: A Phase I/II Therapeutic Strategy for Solid Tumors	Phase I/II	NCT01562626
*Clostridium butyricum*	CBM588	Immunomodulatory Triplet Therapy (CBM588 + Nivolumab/ Ipilimumab) for Advanced or Stage IV Kidney Cancer	phase I	NCT03829111
*Clostridium novyi*	*Clostridium novyi- NT* [123]	Phase I Safety and Tolerability Evaluation of Intertumoral *Clostridium novyi-NT* Spores Formulation in Refractory Solid tumors	Phase I	NCT01924689
				NCT03435952

*GTN* Gestational Trophoblastic Neoplasia, *FIGO* International Federation of Gynecology and Obstetrics*, OSCC* oral squamous cell carcinoma, *MBV* Mixed Bacterial Vaccine, *pLADD* personalized live, attenuated, double-deleted *Listeria monocytogenes*, *CRC* Colorectal Cancer, *PDAC* Pancreatic Ductal Adenocarcinoma, *HPV* Human Papillomavirus, *HER2* Human Epidermal Growth Factor Receptor 2, *NSCLC* Non-Small Cell Lung Cancer.

## Limitations in the intratumoral microbiota research

### Low biomass and contamination risk

Contamination and low biomass are undoubtedly the primary technical challenges. The abundance of microorganisms within tumors is generally low, and their abundance is easily influenced by contamination from other microorganisms or nonbiologically relevant microbial communities. Additionally, decontamination affects bacterial DNA abundance. Although the accuracy of 5R 16S rDNA sequencing and microbial enrichment approaches has increased, it continues to be impaired by sample contamination and storage conditions [7]. Techniques such as host DNA depletion and specialized bioinformatic decontamination (e.g., using QIAamp kits or RIDE procedures) can be applied in an attempt to mitigate these disadvantages [7], but they frequently inadvertently remove true microbial signals while leaving residual host contamination.

### Limitations of sequencing technology

The use of low-cost 16S rRNA gene sequencing for large-scale, low-biomass research has significant limitations. The intrinsic resolution of this method is sometimes limited to the genus level, hiding functionally important strain variation and excluding nonbacterial domains such as those of fungi and viruses [7, 12, 137, 138]. Conversely, whole-genome sequencing (WGS) provides superior resolution at the species/strain level and access to functional potential. However, it is severely hampered by high costs, extreme susceptibility to overwhelming host DNA contamination masking microbial signals, complex data analysis requirements, and insensitivity to very low-abundance communities [7, 12]. This basic methodological discrepancy between 16S and WGS is likely to lead to inconsistent conclusions. Promising computational pipelines such as CSI-Microbes and emerging multimodal approaches aim to overcome these barriers by leveraging single-cell data or integrating multiple data types [7, 139], but they currently lack standardized validation frameworks for solid tumors and face technical challenges in practical implementation.

### Spatial localization issues

Another constraint of current research is the significant variety in microbial distribution inside tumors and their spatial layout, which prevents targeted therapies from efficiently reaching all populations. To address this, researchers may utilize immunohistochemistry (IHC) and fluorescence in situ hybridization (FISH) to investigate the presence and quantity of bacteria in tumors. Live bacteria may be localized using D-alanine techniques and tissue cultures [2]. Ultrastructure may be observed using electron microscopy methods such as CLEM [7]. However, these methods are often inadequate for mapping complex microbial communities at meaningful cellular or subcellular scales within the tumor architecture [2].

New spatial methods, such as invasive adhesion-directed expression sequencing (INVADEseq) and spatial transcriptomics, provide great resolution by combining microbial detection with host transcriptomics at the single-cell or near-single-cell level [140]. However, INVADEseq can detect only the presence of bacteria in the cell and the level of bacterial expression and cannot be used to determine the expression levels of bacterial genes, such as drug-resistance genes. Furthermore, challenges such as host RNA interference and dynamic adjustments to treatment or microenvironment changes remain, and validation across cancer types requires further investigation.

### Reliance on correlative data

Critically, most studies focus on correlation rather than mechanistic exploration. Sample sizes are too small to draw broad conclusions, and heterogeneity in microbial taxonomy and distribution is frequently overlooked. These characteristics also limit statistical power and generalizability, frequently resulting in irreproducible findings across cohorts [138].

Simply expanding the cohort size via meta-analyses is inadequate to resolve this heterogeneity. Instead, longitudinal prospective studies with large samples should be conducted using single-cell sequencing (e.g., SAHMI and INVADEseq), FMT experiments, and machine learning models based on TCGA datasets [6, 7]. Furthermore, single-sample data do not capture the dynamic interactions between microorganisms and the host milieu (e.g., microbial community remodeling in response to therapy), necessitating the use of time-series models [6, 7]. Without such integrated methods, converting microbial correlations into practical treatment options for diverse solid tumors would remain difficult.

### Inability to distinguish causality

Moreover, most studies employ short-term, cross-sectional data, making it difficult to determine whether reported microbial changes are drivers of tumor growth and immunotherapy responses or just the result of a changing tumor habitat [6]. Thus, long-term surveillance and ex vivo experiments are needed [6]. The notion of coevolution between the microbiota and tumor cells, including possible molecular mimicry via tumor-associated antigens (TAAs) and microbial antigens (MoAs), which might guide vaccine design, is yet theoretical and difficult to establish clearly [138].

While experimental strategies such as in vitro microbial pure cultures, tumor organoids cocultured with bacteria (e.g., cervical cancer models) [141], and germ-free (GF) mouse models colonized with specific strains provide pathways for investigating interactions [44], these systems have significant limitations that limit causal inference. Organoids usually lack essential components of the in vivo TME, such as functioning immune compartments, whereas GF mouse models may not completely capture the metabolic complexity and immunological context of human tumors, thereby misrepresenting microbial dynamics and functional effects [142]. Although spatiotemporal systems such as BSCC provide detailed interaction data [7], they oversimplify the complex multicellular environment of solid tumors. Modern methods such as single-cell sequencing (e.g., SAHMI) are useful for identifying correlations, but they primarily provide associative data and cannot directly confirm microbial-driven carcinogenesis or selection forces inside the TME [7].

### Practical barriers to clinical translation

In addition to the technological issues mentioned above, there are further barriers to true clinical translation. For example, patient-specific microbial variability presents challenges in precision medicine. While technological optimization and standardized protocols provide partial solutions, their ability to fully account for profound individual biological differences is unknown, potentially limiting the efficacy of tailored therapy [7]. Furthermore, their in vivo effectiveness is questionable due to inconsistent preclinical results and practical challenges, such as heterogeneous tumor distribution and potential off-target effects [122]. Crucially, broad-spectrum antibiotics, which are often necessary during treatment, disturb the gut microbiota and reduce the efficacy of immunotherapy. This constitutes a confounding factor that is often undercontrolled in translational studies [6]. The translation of medicinal technology is also challenging. Bacterial dosage optimization, treatment response prediction, and combinatorial regimen design are ongoing issues with no recognized clinical frameworks for successful application [6, 143, 144]. Larger, more tightly controlled studies are demanded to determine the therapeutic potential of the intratumoral microbiota.

## Conclusions

The intratumoral microbiota, which is variable in composition and present in various solid tumors, affects the efficacy of immunotherapy. As a crucial component of the TME, the microbiota may alter cytokine secretion and signaling pathways, thereby triggering changes in immune cell function, phenotype, and abundance, which can ultimately affect immunotherapy efficacy in solid tumors. Notably, in addition to well-known intratumoral bacteria, intratumoral fungi also contribute significantly to solid tumor growth. The intratumoral microbiota exerts differential effects on the efficacy of immunotherapy, and these effects are dependent on the microbial species, tumor type, and microsatellite status.

Recent research highlights the curative potential of manipulating the intratumoral microbiota, as microbes can kill cancer cells, enhance antitumor immunity, or serve as drug carriers to improve treatment outcomes. This potential is exemplified by the effectiveness of the BCG vaccine in bladder cancer. Despite promising findings linking the microbiome to ICI efficacy, many unanswered questions remain. Unlike existing literature, which focuses on the heterogeneity of intratumoral microbes and their functional roles in tumor progression or the treatment of specific tumor types, this paper provides a comprehensive synthesis of recent evidence addressing the effects of the intratumoral microbiota on the TME of solid tumors, as well as their clinical translational potential and difficulties, while highlighting the role of intratumoral fungi and the important impact of microsatellite status. The preceding findings provide a theoretical foundation of the precise immunomodulatory pathways by which intratumoral microbes influence solid tumor immunotherapy. This information can facilitate the development of accurate detection and microbe isolation techniques and help address challenges in the clinical translation of intratumoral microbiota-related immunotherapy for solid tumors. This may signal a paradigm shift in oncological treatment techniques.

### Literature search methodology

This section states that our search included peer-reviewed articles from PubMed (2014-2025), using MeSH terms and Boolean operators combining three conceptual clusters: (“intratumoral microbiota” OR “tumor microbiome”) AND (“immune microenvironment” OR “PD-1/PD-L1” OR “CTLA-4”) AND (“immunotherapy response” OR “checkpoint blockade resistance”). Preclinical studies linking microbial signatures to immune cell modulation (e.g., TAM polarization, T-cell exhaustion), clinical cohort analyses correlating tumor microbial profiles with anti-PD-1/PD-L1 outcomes, and meta-analyses quantifying microbial intervention effects (e.g., FMT, probiotics) were included in the literature analysis. Studies that focused primarily on gut microbiota without intratumoral confirmation or that did not include immune profiling (e.g., transcriptomic/flow cytometry data) were excluded from the analysis. Following dual-reviewer filtering, 179 key articles were kept from the original records. The literature search ended in May 2025. This organized technique guarantees that our synthesis focuses on microbial-immune interplay in tumor niches, offering a molecular basis for future translational research.

## Supplementary information

Supplementary Table S1
