# Supplementary material for: Intratumoral microbiota remodeling of the tumor microenvironment impact solid tumor immunotherapy
Source: Cell Death Dis. 2025 Dec 1;17(1):62. doi: 10.1038/s41419-025-08211-w (PMC12828053; doi:10.1038/s41419-025-08211-w)
Supplement: Supplementary file 1 — Supplementary Table S1 [file 41419_2025_8211_MOESM1_ESM.docx]

**Supplementary Table S1:**

| NO. | Abbreviation | Full Term / Definition |
| --- | --- | --- |
| 1 | TME | The tumor microenvironment |
| 2 | PDAC | pancreatic ductal adenocarcinoma |
| 3 | TLSs | Tertiary lymphoid structures |
| 4 | ICI | immune checkpoint inhibitor |
| 5 | GC | gastric cancer |
| 6 | HCC | hepatocellular carcinoma |
| 7 | PC | pancreatic cancer |
| 8 | CEACAM1 | carcinoembryonic antigen-related cell adhesion molecule 1 |
| 9 | PD-L1 | programmed cell death ligand 1 |
| 10 | GVB | gut vascular barrier |
| 11 | STING | Stimulator of Interferon Genes |
| 12 | HNSCC | head and neck squamous cell carcinoma |
| 13 | OSCC | oral squamous cell carcinoma |
| 14 | BC | breast cancer |
| 15 | OC | ovarian cancer |
| 16 | EC | endometrial cancer |
| 17 | NMSC | non‐melanoma skin cancers |
| 18 | TILs | tumor infiltrating lymphocytes |
| 19 | EMT | epithelial-mesenchymal transition |
| 20 | TP53 | tumor protein 53 |
| 21 | BFT | *B. fragilis* toxin |
| 22 | SCC | squamous cell carcinoma |
| 23 | 6-HAP | 6‐N‐hydroxyaminopurine |
| 24 | LPS | lipopolysaccharide |
| 25 | SCFAs | short-chain fatty acids |
| 26 | TIME | the tumor immune microenvironment |
| 27 | DC | Dendritic cell |
| 28 | PAMPs | pathogen-associated molecular patterns |
| 29 | ICD | immunogenic cell death |
| 30 | Treg | regulatory T cell |
| 31 | AHR | aryl hydrocarbon receptor |
| 32 | TMAO | Trimethylamine N-oxide |
| 33 | Tex | T cell exhaustion |
| 34 | *E. coli* | *Escherichia coli* |
| 35 | *Fn* | *Fusobacterium nucleatum* |
| 36 | A2AR | adenosine A2A receptor |
| 37 | NK | Natural killer |
| 38 | ADCC | antibody-dependent cytotoxicity |
| 39 | TAM | tumor-associated macrophage |
| 40 | PRRs | pattern recognition receptors |
| 41 | TLR | Toll-like receptor |
| 42 | c-di-AMP | cyclic di-AMP |
| 43 | *H. pylori* | *Helicobacter pylori* |
| 44 | MDSCs | Myeloid-derived suppressor cells |
| 45 | TANs | Tumor-associated neutrophils |
| 46 | ROS | reactive oxygen species |
| 47 | NE | neutrophil elastase |
| 48 | NETs | neutrophil extracellular trapping networks |
| 49 | PMN | polymorphonuclear leukocyte |
| 50 | PD-1 | programmed death receptor-1 |
| 51 | LUAD | lung adenocarcinoma |
| 52 | HDAC | histone deacetylase |
| 53 | ICB | immune checkpoint blockade |
| 54 | CRC | colorectal cancer |
| 55 | TNBC | triple-negative breast cancer |
| 56 | ESCC | Esophageal squamous cell carcinoma |
| 57 | NSCLC | Non-small cell lung cancer |
| 58 59 60  61 62  63  64 | MSI GTN FIGO  MBV pLADD  HPV HER2 | microsatellite instability Gestational Trophoblastic Neoplasia International Federation of Gynecology and Obstetrics Mixed Bacterial Vaccine personalized live, attenuated, double‐deleted *Listeria monocytogenes* Human Papillomavirus Human Epidermal Growth Factor Receptor 2 |
| 65 | BCG | Bacillus Calmette-Guérin |
| 66 | GBM | glioblastoma multiforme |
| 67 | WGS | whole-genome sequencing |
| 68 | IHC | immunohistochemistry |
| 69 | FISH | fluorescence in situ hybridization |
| 70 | INVADEseq | Invasive adhesion-directed expression sequencing |
| 71 | TAAs | tumor-associated antigens |
| 72 | MoAs | microbial antigens |
| 73 | GF | Germ-free |
